# The E-music box: an empirical method for exploring the universal capacity for musical production and for social interaction through music

**DOI:** 10.1098/rsos.150286

**Published:** 2015-11-18

**Authors:** Giacomo Novembre, Manuel Varlet, Shujau Muawiyath, Catherine J. Stevens, Peter E. Keller

**Affiliations:** 1 The MARCS Institute for Brain, Behaviour and Development, Western Sydney University, Penrith, New South Wales 2751, Australia; 2 School of Social Sciences and Psychology, Western Sydney University, Penrith, New South Wales 2751, Australia

**Keywords:** music box, music production, joint action, universals, neurologic music therapy, non-musicians

## Abstract

Humans are assumed to have a natural—universal—predisposition for making music and for musical interaction. Research in this domain is, however, typically conducted with musically trained individuals, and therefore confounded with expertise. Here, we present a rediscovered and updated invention—the E-music box—that we establish as an empirical method to investigate musical production and interaction in everyone. The E-music box transforms rotatory cyclical movements into pre-programmable digital musical output, with tempo varying according to rotation speed. The user’s movements are coded as continuous oscillatory data, which can be analysed using linear or nonlinear analytical tools. We conducted a proof-of-principle experiment to demonstrate that, using this method, pairs of non-musically trained individuals can interact according to conventional musical practices (leader/follower roles and lower-pitch dominance). The results suggest that the E-music box brings ‘active’ and ‘interactive’ musical capacities within everyone’s reach. We discuss the potential of this method for exploring the universal predisposition for music making and interaction in developmental and cross-cultural contexts, and for neurologic musical therapy and rehabilitation.

## 1. Introduction

Current interest in the universality of music stems from the fact that virtually every human society makes—not just listens to—music [1]. The human predisposition to physically participate with music and to interact with others through music is observable early in life across a range of behaviours. These include infant–carer interactions as well as spontaneous movement to music, which starts in the foetus during the final stages of gestation [2] and develops through infancy [3,4] into more complex forms of action and interactions such as dance and collaborative music making [5].

However, research on musical universality has focused on music perception [6], with musical production and interaction receiving less attention. Furthermore, the relatively scant research on musical production is mostly conducted with musically trained individuals, and is therefore confounded with expertise with a given instrument, genre or musical system, as well as with the amount or effectiveness of musical training. These factors are difficult to control precisely. Therefore, the study of the human predisposition for music making could be revolutionized if the experimenters could bring the training approach, paradigm and exposure under their control. Individuals across a range of ages (infants, children, adults, aged people), cultures, cognitive skills and health conditions could thus be sampled without concerns over needing fine motor control of a conventional musical instrument.

**Figure 1.**
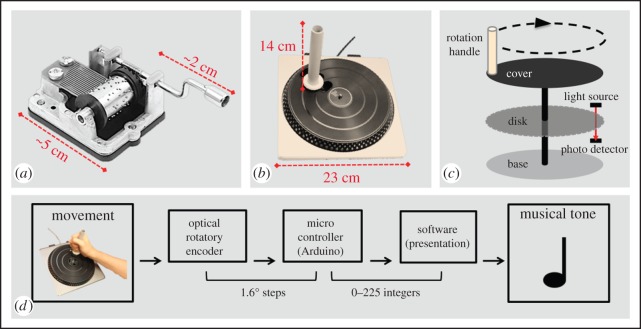
The E-music box. (*a*) Common example of a mechanical music box. (*b*) The electromechanical music box (E-music box) developed for this experiment. (*c*) Diagram illustrating the mechanism of the E-music box. (*d*) Diagram illustrating the hardware and software specifications of the E-music box.

Here, we present an empirical method for studying the human predisposition to make music—and to interact with others through music—in every person, and irrespective of musical training. We rediscovered a popular invention from the nineteenth century—the mechanical music box (figure 1*a*)—and updated it into an electromechanical device (figure 1*b*). The E-music box transforms cyclical rotatory movements into a musical melody whose tempo varies according to the velocity of the rotation. The tunes can be played with correct rhythm and constant tempo by rotating the handle with constant velocity. The E-music box’s digitalization allows for great flexibility in terms of input (movement) and output (music) parameters (which can be pre-programmed; figure 1*d*), and the generation of data with high spatio-temporal resolution. The user’s behaviour is coded both discretely (in terms of the produced effects) and continuously (in terms of the oscillatory movements), and can be analysed using a broad range of linear [7] and nonlinear [8] analytical tools.

The E-music box contributes to a growing arsenal of devices for exploring links between music and movement. Other devices addressing such links have been developed for a diverse range of purposes, such as selecting tracks from a playlist [9], experiencing musical agency [10] or measuring how people move in response to music [11]. Taking a different (but complementary) approach, the E-music box is designed specifically to allow everyone to use movement to make music, and to interact with others through music, as musicians—including ensemble musicians—do.

### 1.1 Proof of principle

**Figure 2.**
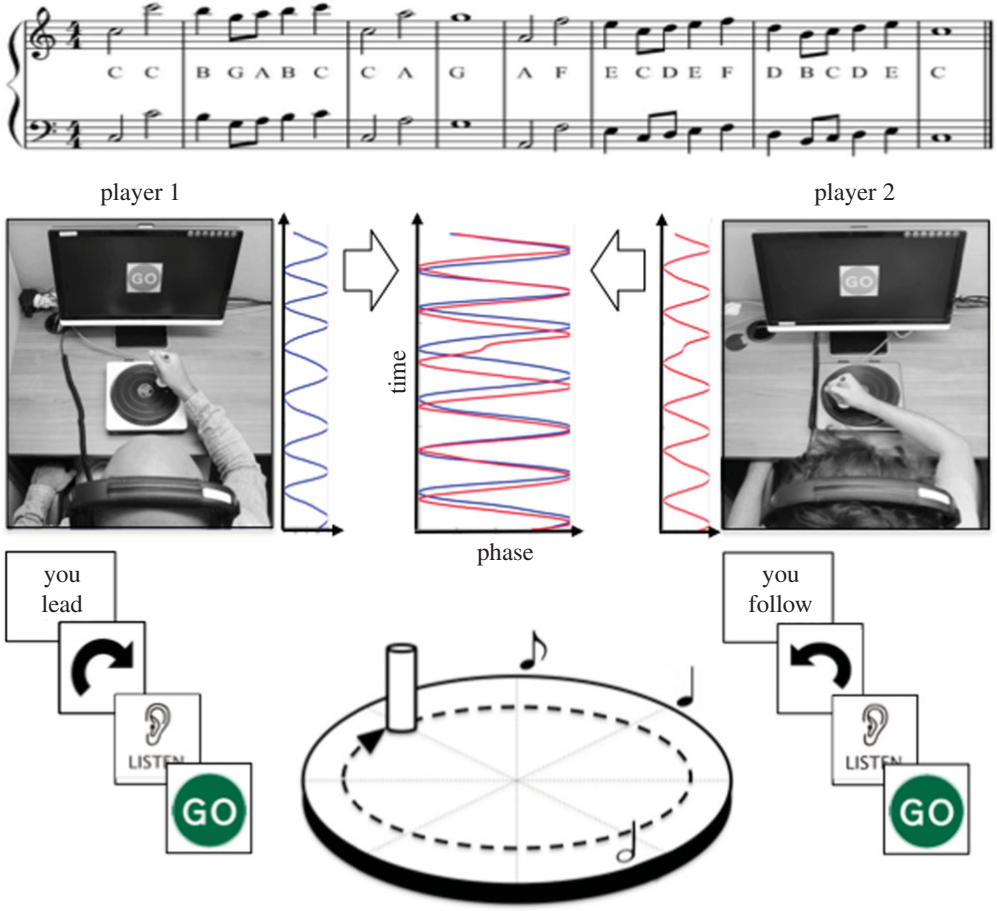
Experimental setting. Two participants (constituting a pair) make music together using E-music boxes. Each participant controls the production of musical tones belonging to one part of the score (either the upper or the lower line, which were higher or lower in pitch, respectively). Depending on the speed of rotation (representative phase time series of the resulting data are plotted), the tempo of the musical part changes accordingly. Rotations of 45°, 90° and 180° are necessary to produce quaver (half beat), crotchet (one beat) and minim notes (two beats), respectively (see bottom part of the figure, middle). The two participants perform the task from separated soundproofed rooms. The monitor provides instructions concerning leadership roles (‘you lead’ or ‘you follow’) and rotation direction (clockwise versus anticlockwise) across trials.

As a proof of principle, we conducted an experiment in which pairs of non-musically trained individuals interacted with one another using E-music boxes. The two players controlled the tempo of two complementary parts from a popular song (‘Somewhere over the rainbow’; figure 2), and synchronized their musical outputs while hearing (but not seeing) each other’s performance.

We tested whether the non-musically trained pairs of participants would interact according to two common musical practices. First, we tested whether the players could intentionally co-perform according to leader–follower roles, resulting in the follower’s musical output lagging behind the leader’s output [12]. Second, we tested the widespread practice wherein lower-pitched voices dominate higher-pitched voices in conveying temporal information. This is commonly observed when bass-ranged instruments lay down the musical rhythm in an ensemble, which is likely to be due to superior time perception for lower musical pitch [13].

To this aim, the two players had control over two identical musical parts that differed only in terms of relative pitch: one player was assigned a higher pitch (high-pitch player) and the other a lower pitch (low-pitch player). Furthermore, the players were asked to synchronize their musical outputs while adopting leadership roles (leaders or followers) or spontaneously (no leader). When leadership roles were assigned, leaders were asked to dictate the overall tempo, and followers were asked to adapt to the leader’s tempo.

To the extent that our method provides a suitable testing ground for exploring universals in joint music making, we made two predictions. First, by computing measures of ‘instantaneous leadership’ (see Methods), we expected followers’ movements and musical outputs to lag behind leaders’ when leadership roles are assigned [12], whereas movements and musical outputs should be well synchronized in the absence of leadership roles. Second, by computing indexes of ‘coupling directionality’ (see Methods), we estimated which player (high pitch versus low pitch) influenced the other’s movements (and resultant musical tempo) to a stronger degree. It was expected that low-pitch players would influence the high-pitch players’ actions to a stronger degree than vice versa.

## 2. Methods

### 2.1 Participants

Sixteen pairs of participants (32 individuals, nine males) were recruited to participate in this study. Sample size was determined based on a small-to-medium effect of *f*=0.2 with *α*=0.05 and *power*=0.95 using G*Power [14], resulting in a required total sample size of nine pairs. Their age ranged between 18 and 41 years (mean 21.69, *s*.*d*.=6.16). None of these participants had ever received musical education or private musical training. All participants were right handed and none had hearing impairments. Ten participants were born in countries other than Australia, including Fiji (*n*=3), Pakistan (*n*=2), Bangladesh (*n*=1), Germany (*n*=1), Thailand (*n*=1), India (*n*=1) and Africa (unspecified nation, *n*=1). Participants’ average level of education was a high school certificate.

### 2.2 Musical material

For this study, we selected the first eight bars from the tune *Somewhere over the rainbow*, originally written for the 1939 movie *The Wizard of Oz* [15]. This tune was chosen for its broad popularity, which was assumed to facilitate the task for the non-musically trained participants. Familiarity with this song was rated 3.74 on average (*s*.*d*.=1.26) on a five-point Likert scale. Four participants who reported being unfamiliar with the song were presented with the original recording once (prior to commencing the experiment). The score of the tune (i.e. section used in this study) is presented in figure 2. The part higher in pitch (upper line in the score) and the part lower in pitch (lower line) were separated by two octaves (i.e. the fundamental frequency of the high-pitch tones was four times greater than the fundamental frequency of the low-pitch tones). The tone and register for the initial notes were C5 (higher-pitch part) and C3 (lower-pitch part). Musical instrument digital interface (MIDI) files were created for each note in the score (using the software Max/MSP, with piano as the musical instrument). Each note had equal duration (500 ms) and loudness (which was controlled by applying a constant velocity of 120). The MIDI files were then converted into wave files for compatibility with Presentation software (see below).

### 2.3 Hardware specifications of the E-music box

Two electromechanical E-music boxes were built by modifying DJ Heros, a low-cost music video game developed by FreeStyleGames to simulate turntables (figure 1*b*). The original circuitry from the turntables was removed and substituted with a microcontroller board (Arduino Leonardo, supplier Sparkfun Electronics) and Connectorized Transmissive Optoschmitt Sensors from Honeywell (HOA7720; supplier Jaycar Electronics) to create a high precision optical encoder. In addition, plastic handles (*length*=14 cm, *diameter*=2 cm) were attached on the turntables cover (6 cm from the centre). The handles were suited for gripping by the hand and were used to rotate the turntables (figure 1*d*). The two optoschmitt sensors, separated by 2 mm, were attached to the rotational platform of the turntable. The sensors were moved over the stationary encoder tooth train to produce two impulse trains phase shifted by ±90° (figure 1*c*).

The resolution of the rotary encoder described above is 0.8°. Output from the rotary encoder is processed by the microcontroller, which converts the pulse trains from the rotary encoder to an angular position with a resolution of 1.6°, which is incremented for clockwise rotation and decremented for counter-clockwise rotation. The output format for the microcontroller is a single byte in binary format (32-bit little-endian integer) counting from 0 to 255 (representing rotational increments of 1.6°). The Arduino board is fitted with a RS232 shield (Cutedigi Electronics, supplier Sparkfun Electronics) to provide a direct connection to the RS232 port on the computer. The serial port on the Arduino is configured with a baud rate of 9600, data rate of 960 bytes s^−1^, data bits—eight with no parity and one stop bit. The Arduino outputs data only when the plastic handle is turned, with maximal temporal resolution of 960 Hz. Although the hardware could support a higher resolution of 0.8°, the custom software (Presentation, Neurobehavioral Inc.) developed for the experiment is limited to receiving a single byte counting from 0 to 255 for efficiency. A detailed set of instructions on how to build an E-music box from a DJ Hero turntable, together with the Arduino code, are provided in the electronic supplementary material. The combined cost of building an E-music box is AUD $50, and all materials are available online (purchases made in 2014).

The E-music box could of course be built using different technology or hardware from ours. Yet, we believe that our implementation is very efficient to satisfy the conditions of (i) outputting data with high temporal and spatial resolution, (ii) minimizing costs, and (iii) relying on a movement that is simultaneously: (a) simple, natural and commonly widespread (in this case, similar to stirring a large spoon), (b) involving numerous joints and muscles (in this case, the upper limbs, resulting in a physically healthy activity that suits rehabilitation scenarios, see below) and (c) constrained in its trajectory (in this case, restricted by the trajectory of the handle) in order to obtain more controlled (i.e. less variable) data. For a discussion of why custom-built devices are generally preferable to commercially available solutions for empirical research, the reader is referred to Ravignani *et al.* [16].

### 2.4 Software specifications of the E-music box

The outputs from the two E-music boxes were fed into a high-performance Dell computer (Dell OptiPlex 960, with dual 3.0 GHz Xenon processors) via two separate serial ports. The computer was equipped with Presentation software, which was used for the delivery of the musical tones (as well as for controlling the experiment, see below). Presentation was programmed to collect the codes received from each E-music box, and to present series of WAV files corresponding to the two musical parts displayed in the score in figure 2. The codes received from one E-music box controlled the presentation timing of the tones belonging to only one musical part. Tone presentation timing depended on the succession of codes received as well as on the specific duration of each note specified in the musical score. In particular, it was necessary to rotate the E-music box handle by 45° in order to play a quaver, or eighth note (i.e. half a beat), by 90° for a crotchet, or quarter note (one beat) and by 180° for a minim, or half note (two beats; for a schematic illustration, see figure 2, bottom part, middle). This implied that, as long as the E-music box was rotated at constant velocity, the outputted music would preserve its metrical structure. This aspect, which was inspired by the mechanics of the original music box (figure 1*a*), was important in order to permit non-musically trained participants to produce metrically correct musical tunes. Presentation recorded the degree codes received from the E-music boxes, the codes’ timing and stimulus presentation.

### 2.5 Procedure

The two participants constituting a pair operated the E-music boxes from two separate soundproofed booths and therefore had no visual contact (figure 2). Each participant was seated at a desk equipped with a computer monitor, an E-music box and headphones (Sennheiser HD 280 pro).

Participants were instructed to rotate the E-music box with the right hand, and to synchronize their musical output with the musical output of their partner. They were told to synchronize, as accurately as possible, as if they were ‘singing a song together’. As a secondary goal, participants were asked to adopt leadership roles as requested (see below). Specifically, leaders were instructed to play at a pace that was at the same time comfortable for them, and achievable for the followers, who instead were instructed to adapt to the tempo of leaders. If ‘no leadership’ was requested, participants were asked to achieve synchronization spontaneously.

Each trial began with the visual presentation (on the computer monitor) of a fixation cross (500 ms), followed by a circular arrow (3000 ms). The arrow could either point in a clockwise or anticlockwise direction, which was meant to instruct the participants how to turn the E-music box handle during the trial. Next, a metronome playing four beats (750 ms intervals) was presented via headphones, while the monitor displayed an ‘ear’ symbol accompanied by text stating ‘listen’ (figure 2). When the metronome ended, a ‘go’ sign was presented on the monitor to indicate to start rotating the E-music box handle. From this point, by rotating the E-music box handle, each participant had control over the timing of the tones belonging to the musical part they were assigned to. Importantly, movement phase was coded relatively to the handle position at trial onset (i.e. the phase was set to zero when the ‘go’ sign was presented). The first outputted musical tone (consisting of a minim note; figure 2) was presented after a 45° rotation (in the instructed direction), regardless of the handle’s absolute position at trial onset (subsequent notes were produced following rotations of 45°, 90° or 180° depending on rhythmic duration, see above). The participants could always hear their musical output mixed with their partner’s output. A trial ended when both musical parts had been played entirely. At that point, the ‘go’ sign disappeared, and a new trial began (after a 500 ms intertrial interval).

All the pairs performed 64 trials, which were grouped into eight blocks. Each block comprised four trials during which the two participants turned the E-music box in the same direction and four in opposite directions. The assignment of directions was counterbalanced across participants. Leadership instructions were provided at the outset of each block, and remained unchanged for the whole block. These instructions were provided by presenting text on the monitor stating either: ‘you lead’ and ‘you follow’ (four blocks, with alternated instructions across participants) or ‘no leadership’ (four blocks, with the same instruction for both participants). The order of the blocks and the trials within each block was randomized. To familiarize participants with the procedure, each pair performed a few training trials before starting the experiment until they felt clear about the task. During this practice, it was particularly important to allow each participant to autonomously produce the part they were assigned to (higher or lower in pitch). This was crucial to permit the participants to discriminate self- versus other-produced musical outputs. For this reason, participants were randomly assigned to either the part lower or higher in pitch from the start of the practice, and this remained unchanged for the whole experimental session (which lasted around 40 min).

### 2.6 Data analysis

**Figure 3.**
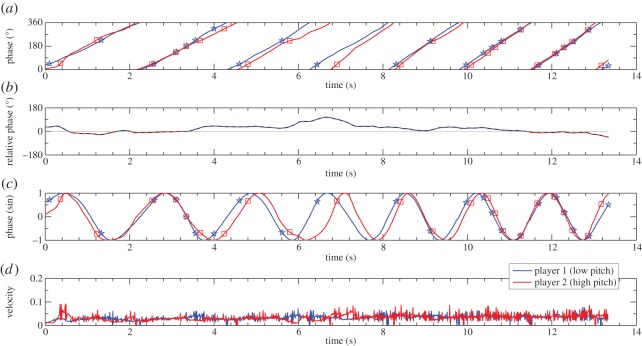
Real-time movement dynamics of two E-music box players. The figure shows representative data from one trial. The blue line indicates the rotatory movements performed by the low-pitch player, and the red line indicates the movements performed by the high-pitch player. (*a*) Phase expressed in degrees (between 0 and 360); (*b*) relative phase (the colour indicates which player’s movements are produced earlier); (*c*) phase expressed in sine; and (*d*) rotation velocity. Markers indicate when the musical tones are produced.

The collected data consisted of time series representing the onset time of the musical tones and the position of the E-music box handles (in degrees) within each trial. Representative data from one trial are provided in figure 3*a*,*c*, where the (rotatory) movement phases of each E-music box and the resultant musical tones are plotted as a function of time. These data were used to examine three properties of the interpersonal coordination: instantaneous leadership, coordination precision and coupling directionality. For each of these properties, two different measures were calculated.

#### 2.6.1 Instantaneous leadership and coordination precision

The instantaneous leadership measures were intended to index which participant (high-pitch or low-pitch player) was lagging behind in the interpersonal coordination. We computed this index both discretely and continuously, i.e. at the level of the actual tones produced (lag of the discretely produced musical tones) as well as the movements employed to produce the musical tones (lag of the continuous rotatory movements).

Discrete data (onset time of the musical tones) were analysed consistently with sensorimotor synchronization research employing finger tapping or piano performance tasks [17,18]. Accordingly, we subtracted the onset time of each tone belonging to the low-pitch part from the complementary tone belonging to the high-pitch part. Next, we calculated the mean and the standard deviation of the computed signed asynchronies within each trial. Mean signed asynchronies indexed the instantaneous leadership. More specifically, positive mean asynchronies indicated that low-pitch tones were produced overall earlier than the high-pitch tones, while negative values indicated the opposite. The standard deviation of the asynchronies, on the other hand, indexed the precision of the coordination. The smaller the standard deviation, the more precise (i.e. less variable) is the coordination [18].

Continuous data, i.e. time series indicating the angular position of each E-music box handle in 1.6°-steps, were analysed consistently with previous research that examined interpersonal synchronization of oscillatory movements using continuous measures of relative phase [19]. Because the angular position of each E-music box handle was coded (in Presentation software) only when the handle was turned, sampling rate was not regular. Therefore, the positional time series were interpolated to form uniform time intervals of 10 ms (i.e. 100 Hz sampling rate), and all movements were converted into clockwise-like movements. The continuous phase of each participant corresponded to the angular position of his or her E-music box handle with respect to the position held at trial onset. Continuous relative phase was then computed by subtracting the unwrapped^1^ continuous phase of the high-pitch player from the unwrapped continuous phase of the low-pitch player [8] (shown in figure 3*b*). Accordingly, positive and negative relative phase values indicated that low pitch and high pitch led the coordination, respectively. Similar to the asynchrony data, we computed mean and standard deviation of the relative phase within each trial, to assess the instantaneous leadership and precision (i.e. variability) of the coordination.

#### 2.6.2 Coupling directionality

The continuous phase time series were also used to estimate the directionality of the coupling within each trial, i.e. which participant (high-pitch or low-pitch player) in a pair was relatively more influential, and which one was more adaptive. For this purpose, we computed two indices.

First, we estimated the directionality of the coupling between participants using the evolution map approach proposed by Rosenblum & Pikovsky [20], also used in interpersonal coordination dynamics [21]. This method quantifies the directionality of the coupling between two self-sustained oscillators by estimating the ratio of the coupling terms from the phase (unwrapped) time series of the movements. This method provides a directionality index, signified *d*(1,2), which can vary from 1 to −1 (where 1 and −1 are the cases of unidirectional coupling, and 0 is the case of a perfectly symmetrical bidirectional coupling). Here, a positive value indicated higher influence of the low-pitch player over the high-pitch player, while a negative value indicated the opposite.

Second, we computed an additional measure indexing the directionality of the coupling, which was based on the assumption that leaders would be more regular in their movements, and followers more variable. Specifically, increasing movement variability was assumed to reflect the increasing adaptation (i.e. use of error correction) to achieve sensorimotor synchronization [22]. To compute this measure, we first estimated the velocity of rotation (separately for each participant) by computing the first-degree derivative of the unwrapped phases (figure 3*d*). This measure indicated the real-time relative movement velocity within a given trial (which increased and decreased according to the task’s demands; compare figure 3*a* and *c* with *d*). Next, we calculated the standard deviation of each participant’s velocity as an index of variability. Finally, we subtracted the low-pitch player variability from the high-pitch player variability to estimate whose velocity was more variable. This difference value, which we termed ‘relative timing variability’, was positive if the high-pitch player was more variable (i.e. more adaptive) than the low-pitch player, and negative if the low-pitch player was the more variable. Hence, positive relative timing variability indicated that the low-pitch player was influencing the high-pitch player more than vice versa.

### 2.7 Statistics

All dependent measures were analysed following the same procedure. First, trials during which one of the participants began rotating the handle in a direction different to the one instructed (during the first 2000 ms following the ‘go’ sign) or whose coordination index deviated more than 3 s.d. from the (participant-specific) mean were discarded (2.9% and 1.7% of the raw data, respectively). Next, condition-specific averages were computed separately for each pair of participants. A preliminary analysis determined that our dependent measures did not change as a function of whether the two participants were rotating the E-music box in the same direction (both clockwise or anticlockwise) or in different directions (one clockwise and the other anticlockwise). Therefore, these data were collapsed and entered into repeated measures ANOVAs with one within participants factor (leadership) having three levels: high-pitch leads, no leader, low-pitch leads.

## 3. Results

Our dependent measures of interpersonal coordination consisted of two indices of instantaneous leadership (mean signed asynchronies and relative phase), two indices of precision (standard deviation of the signed asynchronies and standard deviation of the relative phase) and, finally, two indices of coupling directionality (*d*(1,2) and relative timing variability; see ‘Data analysis’ section and figure 3).

### 3.1 Instantaneous leadership

**Figure 4.**
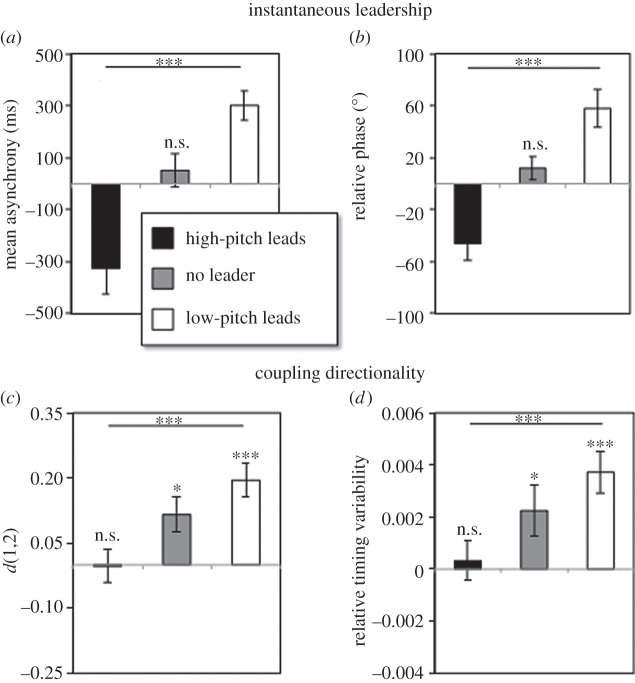
Interpersonal coordination results. (*a*) (Mean signed asynchronies) and (*b*) (mean relative phase) are indices of instantaneous leadership. (*c*) (*d*(1,2)) and (*d*) (relative timing variability) are indices of coupling directionality. Positive values indicate that the low-pitch player produces musical tones and movements earlier than the high-pitch player (in (*a*,*b*)), or that the low pitch player was influencing the high-pitch player performance more than vice versa (in (*c*,*d*)), whereas negative values indicate the opposite. Each separate bar represents a different leadership condition. Error bars indicate 1 s.e.m. n.s., not significant, *p<0.05, ***p<0.001.

Our measures of instantaneous leadership indexed which player (high-pitch or low-pitch) was lagging behind across conditions. These measures included mean signed asynchronies and mean relative phase, which can be seen in figure 4*a* and *b*, respectively. The ANOVAs on these data yielded a main effect of leadership for both the signed asynchronies (*F*_2,30_=13.84, *p*<0.00001) and the relative phase (*F*_2,30_=18.51, *p*<0.00001). These results indicated that instructed followers (both high-pitch and low-pitch players) produced their musical output slightly after the leaders (around 300 ms after, on average), and their E-music box handle lagged behind leaders’ handles (around 50° behind, on average). This result was further supported by significant one-sample *t*-tests comparing all high-pitch leads and low-pitch leads conditions versus zero (all *ps*<0.01).

During the no leader condition, the overall synchronization approached 0 ms asynchronies and zero relative phase degrees. This was confirmed by two separate one-sample *t*-tests comparing the asynchronies (*t*_15_=0.865, *p*=0.401) and the relative phase (*t*_15_=1.34, *p*=0.197) from the no leader conditions versus zero. This suggested that, when no leader was assigned, high- and low-pitch players were presumably involved in a state of instantaneous co-leadership.

### 3.2 Coordination precision

Our measures of coordination precision (see data in table 1) did not yield significant effects of leadership, neither for the standard deviation of the signed asynchronies (*F*_2,30_=1.097, *p*=0.347) nor the standard deviation of the relative phase (*F*_2,30_=2.602, *p*=0.091). The relative phase data, however, showed a trend towards significance. This was indicative of an overall increase in precision in the no leader condition compared with the conditions with a designed leader. Indeed, a planned comparison testing for the quadratic trend of the factor leadership (high-pitch and low-pitch versus no leader) reached significance (*F*_2,30_=7.325, *p*=0.016). Overall, these results indicated that, although precision tended to be higher in the no leader condition compared with the high-pitch and low-pitch conditions, the latter two were not different from one another. Thus, coordination precision did not change depending on whether low-pitch or high-pitch players led.

**Table 1.** Measures of coordination precision. Mean and standard deviation of the signed asynchronies and relative phase.

	high-pitch leads	no leader	low-pitch leads
asynchronies (s.d.)	502.32±154.70	466.18±179.14	456.99±131.38
rel. phase (s.d.)	77.30±26.62	68.16±26.90	72.07±21.64

### 3.3 Coupling directionality

The measures of coupling directionality—*d*(1,2) and relative timing variability—are displayed in figure 4*c* and *d*, respectively. The ANOVAs on these data yielded main effects of leadership for both *d*(1,2) (*F*_2,30_=13.197, *p*<0.0001) and relative timing variability (*F*_2,30_=15.704, *p*<0.0001). These results indicated that leadership instructions changed the directionality of the coupling between participants.

As predicted, both measures revealed an asymmetry in the coupling between low-pitch and high-pitch players. More specifically, these data indicated that—overall—low-pitch players influenced high-pitch players more than vice versa. This was demonstrated by *t*-tests contrasting each leadership condition versus zero. These tests revealed that, when the low-pitch players were leading, the *d*(1,2) (*t*_15_=5.147, *p*<0.001) and relative timing variability (*t*_15_=4.61, *p*<0.001) were significantly different from zero. This occurred also when no leadership roles were assigned (*d*(1,2): *t*_15_=2.85, *p*=0.012; relative timing variability: *t*_15_=2.30, *p*=0.036), but importantly, not when the high-pitch player was leading the coordination (*d*(1,2): *t*_15_=0.054, *p*=0.95; relative timing variability: *t*_15_=0.43, *p*=0.67).

Taken together, these results indicated that the coupling directionality was overall asymmetrical. Low-pitch players influenced high-pitch players to a stronger degree than vice versa, even when no leadership roles were designed. This suggests that controlling a relatively lower musical pitch led to stronger interpersonal influence over the partner.

## 4. Discussion

We presented a rediscovered and updated invention—the E-music box—that provides an empirical method to study the universal human predisposition to make music and to interact with others through music. Reinventing the wheel, the E-music box has the potential to revolutionize the scientific study of musical production by enabling rigorously controlled investigations in every person: across development, cultures, health conditions and irrespective of musical training.

In the following sections, we first discuss the results of the experiment we conducted (as a proof of principle) to demonstrate the potential of the E-music box to study musical interaction between individuals who never received musical training. Then, we discuss two research areas that would benefit from this empirical method: (i) the ontogenetic and phylogenetic origins of musical behaviour, and (ii) clinical interventions that use music for rehabilitation and therapy.

### 4.1 Proof of principle

A proof-of-principle experiment was conducted to assess the suitability of the E-music box to explore musical production and interaction, irrespective of musical training. To this aim, pairs of untrained participants performed a musical melody in ensemble (while hearing, but not seeing, each other) using E-music boxes.

By manipulating leadership roles (leader and follower) and relative pitch (one higher, one lower) of the interacting players, we observed two widespread musical practices. First, the players were able to take leader and follower roles when requested, which involved followers’ outputted music and movements lagging behind leaders’, as commonly observed in musical ensembles [12]. Furthermore, the participants were able to maintain near-perfect synchronization of the musical sounds when asked to synchronize spontaneously (no leader condition). This result is important because it demonstrates that non-musically trained individuals are potentially able—using this method—to achieve interpersonal synchronization during joint music making.

Second, besides addressing synchronization, our analyses explored to what extent the two players were able to mutually influence and adapt to each other’s actions. This was investigated using measures of coupling directionality. Consistent with the common musical practice where bass-ranged instruments lay down the musical rhythm [13], we observed an asymmetry in coupling directionality across high- and low-pitch players. Essentially, controlling a relatively lower musical pitch led to stronger interpersonal influence over the partner than vice versa, and this was observed even when none of the players was designed as leader.

These results highlight the potential of our empirical method to investigate musical interaction beyond musically trained individuals. Specifically, our proof of principle was successful in demonstrating that generally joint music making (implying synchronization and mutual adaptation), and particularly certain musical practices (such as leadership and lower-pitch dominance), can be effectively studied in the general population using our method.

### 4.2 Exploring the universal predisposition for music making and musical interaction

It is widely accepted that musical behaviour is a human universal. Yet, music’s origins and functions are mysterious. Theories span from music being a signal (a prelinguistic communication system [23]), to a tool to establish social bonds and group cohesion [24]. Similarly, research on human (ontogenetic) development suggests that music is largely a social endeavour [25]. When music and dance are social parts of devotional or ritual activity, they are participatory, with the possibility for all members to contribute in different ways: singing, playing an instrument, stamping one’s feet [26] or verbally interjecting [27]. Indeed, Malloch & Trevarthen [28] theorize that, from infancy, the core of human communication and companionship is ‘communicative musicality’ [28].

Despite the important social and interpersonal functions played by music across societies, the empirical study of musical universality has mostly focused on perception, such as perceived emotion [29] or emotion–movement correspondences [30]. Remarkably, musical production—without which there is no music to be perceived—and even more so musical interaction have been substantially neglected, perhaps owing to the confounding effects of expertise.

Here, we argue that the E-music box, by permitting every person to make familiar or unfamiliar, relatively intricate music and to interact with others through music, fills a gap in research. Indeed, given that the roots of music production are much older and that evolutionarily significant musical forms are communal and participatory, a musical device that enables joint music making without the need for extensive practice serves a significant research need. Moreover, a device that produces sounds that are not culturally constrained but able to be programmed to produce a range of timbres, scales and tunings is an asset for studying culturally diverse materials, populations, and in fieldwork settings.^2^ This enables rigorously controlled experiments that might eventually prove powerful in shedding light upon the mysterious ontogenetic [25] and phylogenetic [31,32] origins of music.

### 4.3 The E-music box for neurologic therapy and rehabilitation

Music-based movement activities have been successfully used as a complementary (low-cost) rehabilitation approach for treating acquired or congenital brain disorders, particularly those associated with motor dysfunctions [33]. These include gait-related activities in Parkinson’s disease [34] and stroke rehabilitation (including aphasia) [35].

However, music procedures that involve actual music making are scarcely used because they often require training and can only target patients with moderate impairments [36,37]. Here, we propose that the introduction of the E-music box in these therapeutic approaches could be particularly fruitful. Without undergoing any training procedure, a patient could play rewarding (i.e. patient-selected, [38]) musical tunes. Furthermore, input (movements) and output (music) parameters could be pre-programmed according to each patient’s needs. This could be a considerable advantage and eventually extend neurologic musical therapy to an easily accessible music making procedure that could be applied to a larger patient population than before.

The E-music box could introduce an interactive (yet very controlled) component in these procedures. The patients, besides making music, could possibly interact with another individual through music. Because the musical movements are coded as continuous oscillatory data, the entrainment between music players can be estimated using a range of powerful analytical tools—including linear and nonlinear techniques—that are suitable for assessing different aspects of performance [39,40]. This scenario could be particularly useful for traditional patient–therapist music therapy procedures and provide a methodologically sound method for testing the origins of its beneficial effects [41,42]. Furthermore, in the absence of a therapist, a computer-controlled virtual partner could operate the (interactive) E-music box (cf. [43,44]), and the degree of adaptivity of such a virtual partner could be progressively regulated according to the patient’s needs [45,46].

Finally, because pairs of E-music boxes can be used for studying interpersonal human interaction (as demonstrated here), this method is also likely to hold potential for understanding (and perhaps treating) social disorders where conventional communication may be impaired, such as autism [47]. This suggestion is reinforced by evidence indicating spared or enhanced musical abilities in autistic children [48], and musical interaction promoting empathic behaviour [49]. The call for novel interactive methods to study autism spectrum disorder [50], particularly musical interventions [51], is therefore answered by development of the E-music box.

## 5. Conclusion

We established an empirical method—the E-music box—to study the universal human predisposition for music making, and for interacting with others through music. The E-music box does not require training in order to be operated. Furthermore, its input (movement) and output (music) parameters can be flexibly pre-programmed. The E-music box thus permits virtually every person to produce diverse music and to interact with others through music in a controlled scenario. This opens interesting perspectives for research on the universal predisposition for music making, musical communication and musical interaction across ages (from infancy to elderly) and cultures. Moreover, this method might further develop the use of music for therapeutic and rehabilitation purposes, by bringing ‘active’ and ‘interactive’ musical practices within everyone’s reach.

## Supplementary Material

SI_1_ARDUINO_usercode_.pdf

## Supplementary Material

SI_2_Instruction for building a music box using a DJ hero_.pdf

## Footnotes

1 Phase unwrapping involves adding/subtracting 2*π* radians each time a discontinuity between 2*π* radians and zero radians (i.e. increasing after 360°, or decreasing after 0°) is detected (see [8]).

2 Besides providing more flexibility in overcoming constraints related to interindividual, intercultural or interage differences, it is also worth pointing out that the E-music box is a readily accessible method from the perspective of both the user (simple task) and experimenter (simple equipment to set up and no sophisticated analytical skills required).

## Data accessibility

Data are available from the Western Sydney University institutional repository: http://dx.doi.org/10.4225/35/561380fc38404.
