# Supplementary material for: The E-music box: an empirical method for exploring the universal capacity for musical production and for social interaction through music
Source: R Soc Open Sci. 2015 Nov 18;2(11):150286. doi: 10.1098/rsos.150286 (PMC4680608; doi:10.1098/rsos.150286)
Supplement: SI_1_ARDUINO_usercode_.pdf [file rsos150286supp1.pdf]

```
char R;
int val;
 int encoderPinA = 3;
int encoderPinB = 2;
float encoderPos = 0;
int encoderPinALast = LOW;
int encoderPinBLast = LOW;
int n = LOW;
int m = LOW;

void setup() {
  pinMode (encoderPinA,INPUT);
  pinMode (encoderPinB,INPUT);
  Serial1.begin (9600);
}

void loop() {
  n = digitalRead(encoderPinA);
  if ((encoderPinALast == HIGH) && (n == LOW))
  {
    if (digitalRead(encoderPinB) == LOW)
    {
      encoderPos = encoderPos + 1 ;
    }
    else
    {
      encoderPos = encoderPos - 1;
    }
   if(encoderPos > 224 )
    {
       encoderPos = 0;
    }
    if (encoderPos < 0)
    {
      encoderPos = 224;
    }



    Serial1.write(int(encoderPos));

  }
```

```
  encoderPinALast = n;


  if (Serial1.available()>0)
  {
    R = (char)Serial1.read();
    if (R == 'r' || R == 'R')
    {
      encoderPos = 0;
    }
  }

  if (Serial.available()>0)
  {
    R = (char)Serial.read();
    if (R == 'r' || R == 'R')
    {
      encoderPos = 0;
    }
  }

}
```
