# Supplementary material for: The E-music box: an empirical method for exploring the universal capacity for musical production and for social interaction through music
Source: R Soc Open Sci. 2015 Nov 18;2(11):150286. doi: 10.1098/rsos.150286 (PMC4680608; doi:10.1098/rsos.150286)

Instruction for building a music box using a DJ hero

1. Remove the back cover

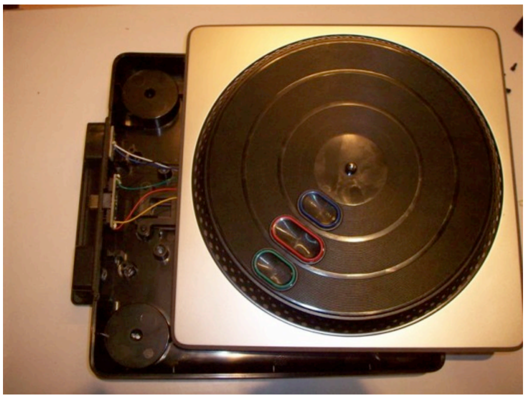

2. Remove the switch, usb connector and the locking mechanism.
3. Cut the wires at the base of the circuit boards.

Cut the wires as close to the circuit board as possible

Remove the locking mechanism, connector and the reset switch

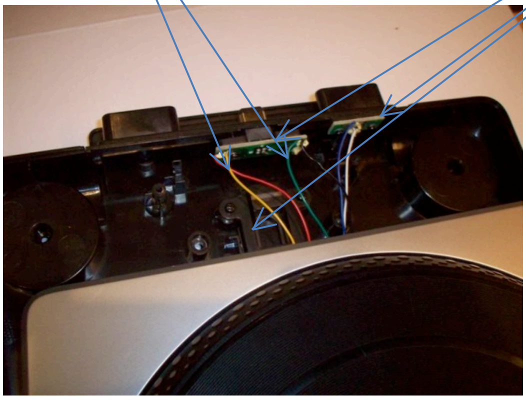

4. Remove the 4 screws holding the turntable to the cover

Remove the 4 crews holding the turntable

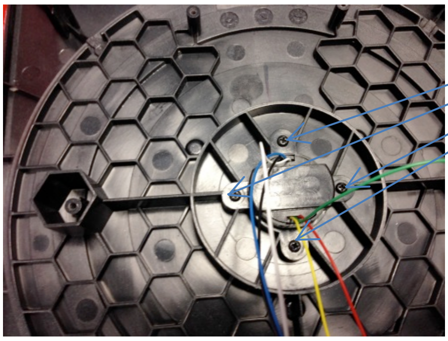

5. Remove the cover of the turntable

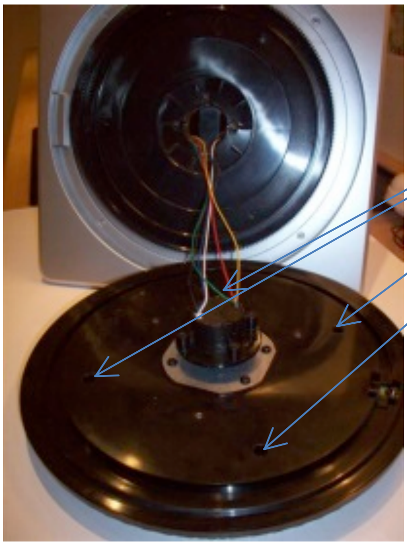

Remove 4 screws to remove cover

6. Cut the wires at the base of the circuits and remove the circuits and the 3 switches

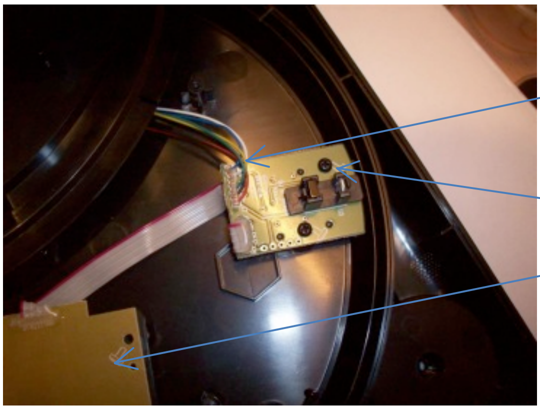

Cut the wires as close to the circuit as possible

Remove the screws and the circuit boards and switches

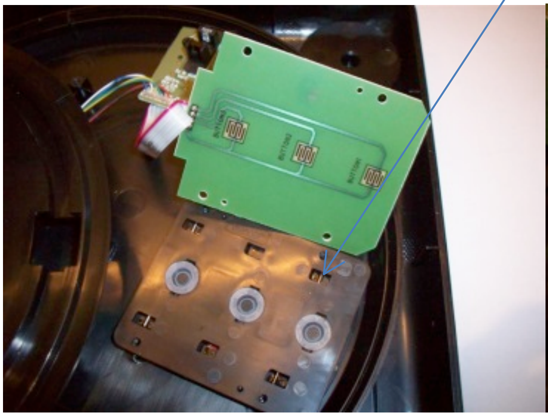

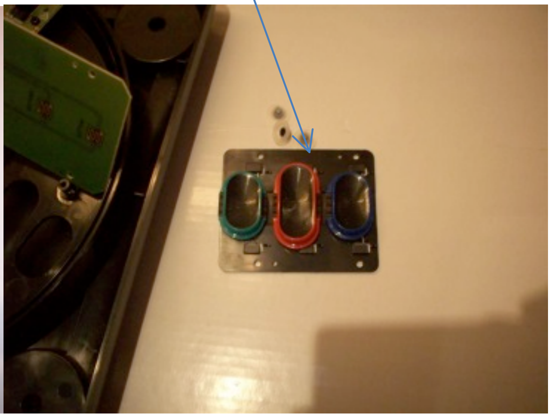

7. To fix the new sensors remove the plastic bits that are in the way and enlarge the groove that holds sensors.

Remove plastic bits in the way of the sensors

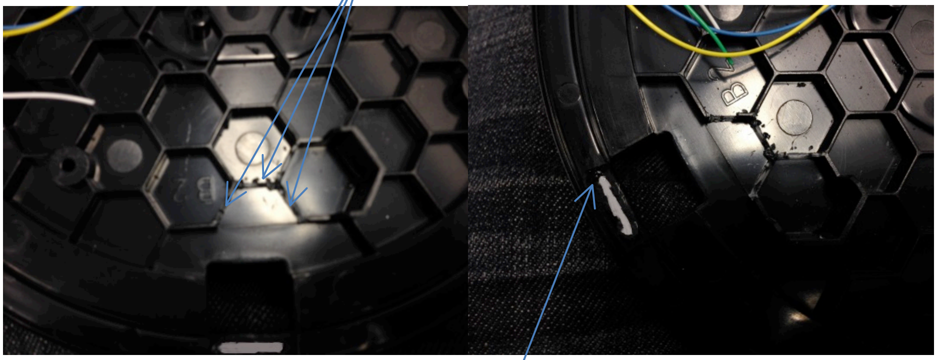

Enlarge the grove that holds the sensors

8. Fix one senor as shown at the far end of the grove by drilling a hole and using a screw and nut.

Drill the first hole to hold the sensor in place, and screw the sensor

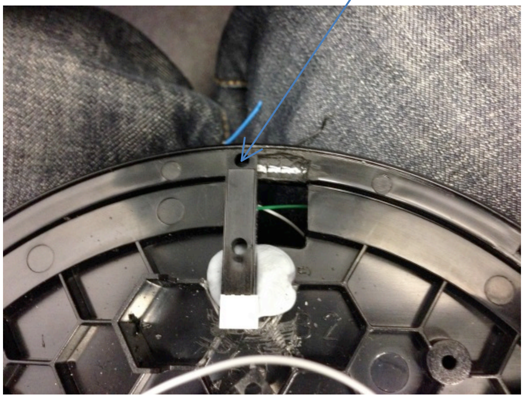

Connect the wires to the connectors. Red and white - positive 5, yellow and blue - signal, black and green - ground

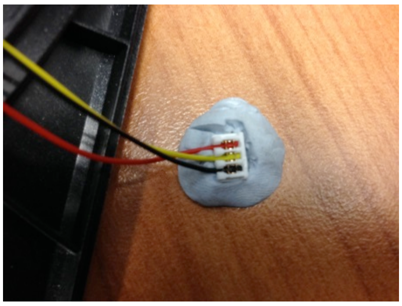

9. Place the second sensor in place using blu tack such that the output from the sensors is seen on the scopes as shown.

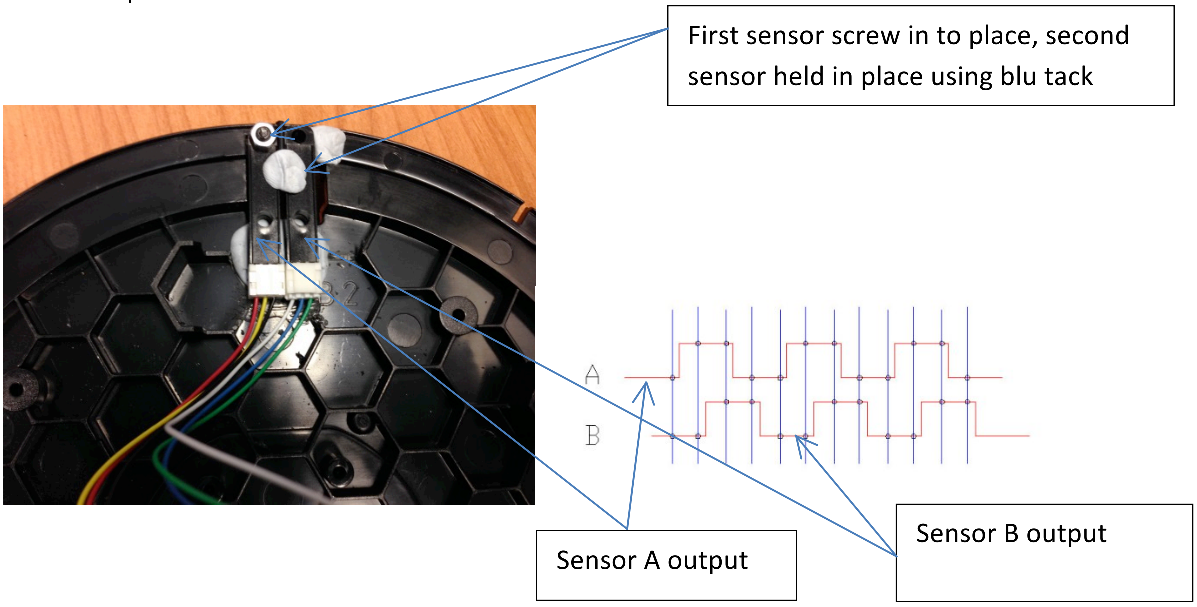

10. Now drill the second hole and screw in the sensor.

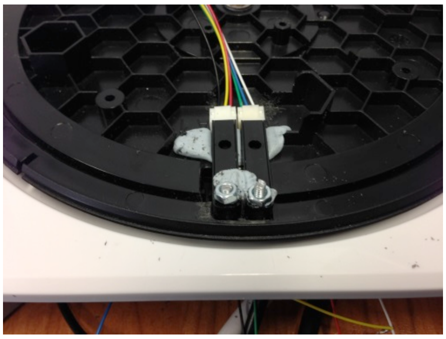

11. Cut the disc spindle such that it fits easily in to the second opening on the cover of the turntable

Disc spindle cut to fit the cover

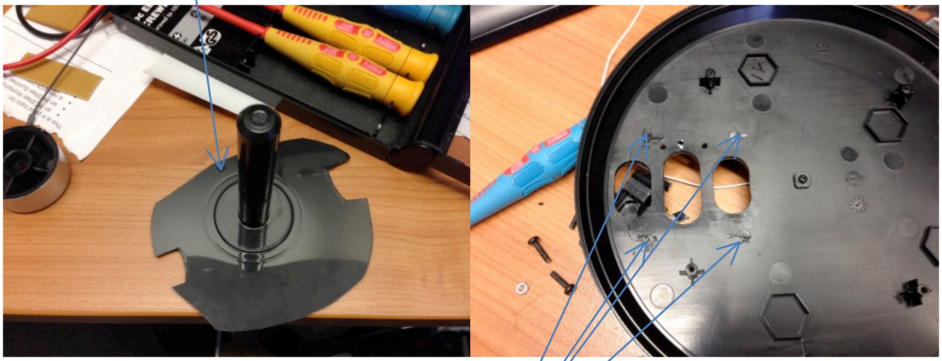

Some plastic removed to fit the disk spindle

12. Drill holes and fit the disk spindle in place using four screws.

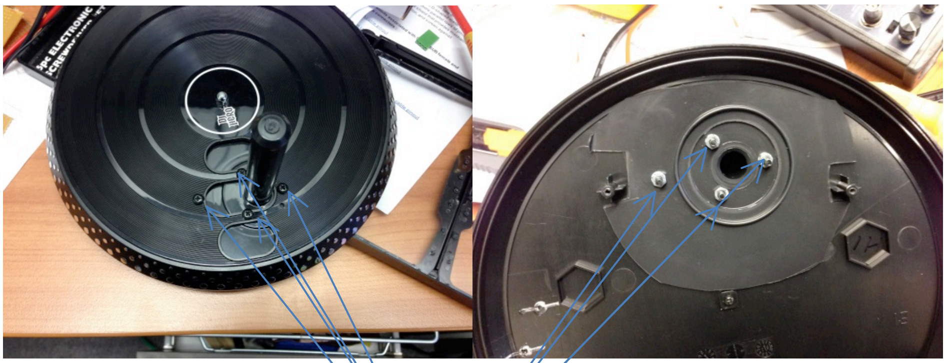

Disk spindle held in place using four screws.

13. Reassemble the turntable and base, if the screws holding the sensor touch the plastic bit shown in figure remove it.

The plastic bit to be removed

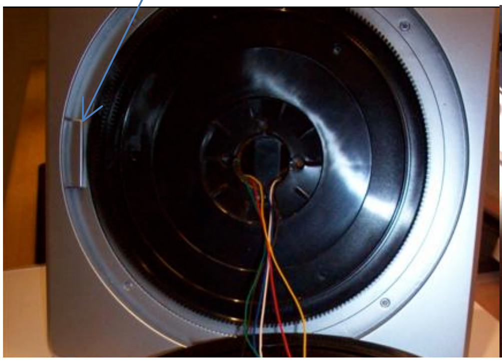

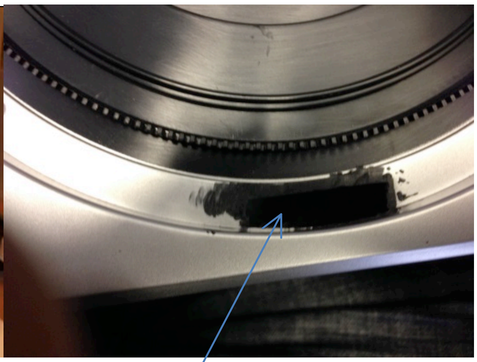

Plastic bit removed

14. Solder the wires from the turn table to a vero board and connect the cable that takes the signal to the micro controller to the vero board and screw the vero board the screw holes of the locking mechanism. There is no circuit this is just to extend the cable to reach the microcontroller.

Wires from the sensors and the microcontroller connected to the vero board.

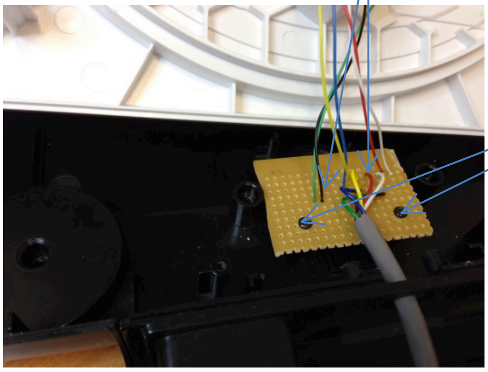

Screws to hold the vero board to the base plate

15. Assemble the serial port shield.

Solder the connectors to the board

Connector in place for soldering

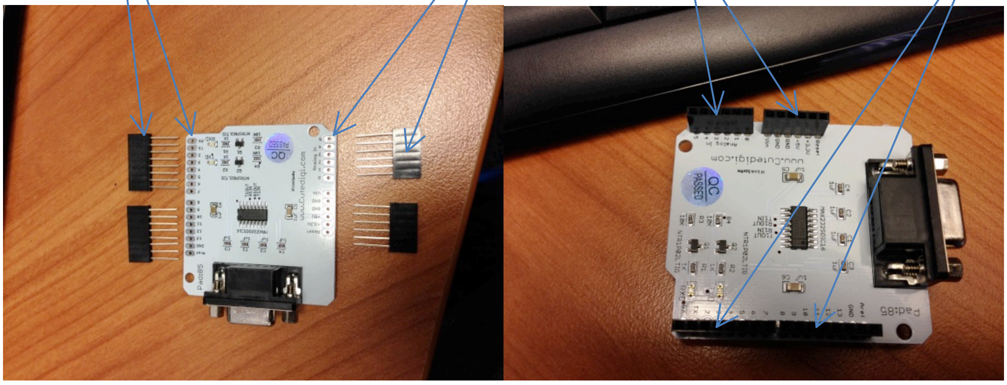

Soldered connectors

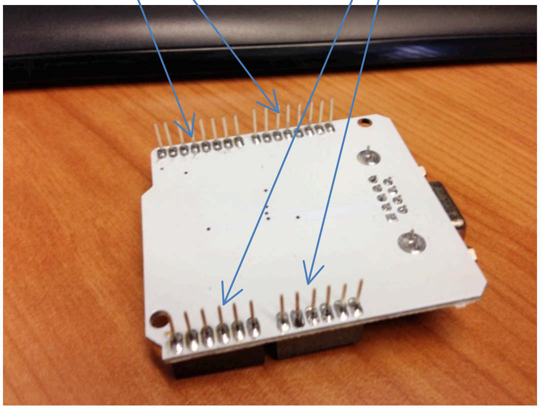

16. Build a board to connect the cable from the sensors to the micro controller

2 pins used for connecting sensors to the microcontroller

Separate the signal side and the power side

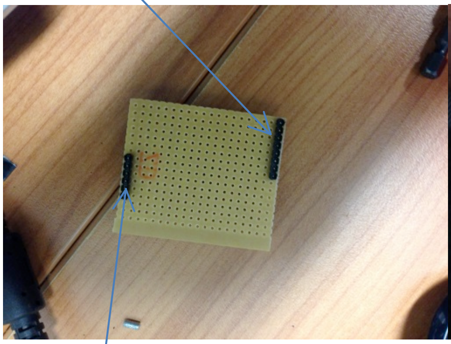

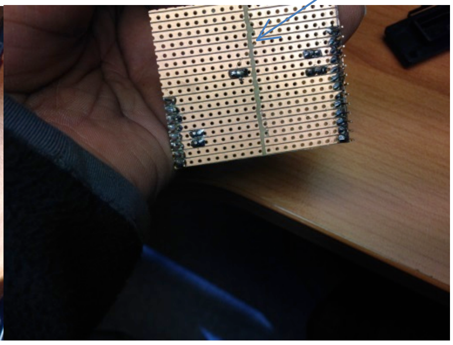

Power and ground connection

17. Connect the cable from the sensor to the board

Ground

5V+

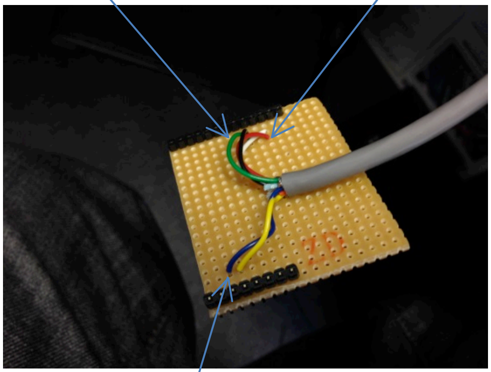

Signal pin 2 and pin 3

18. Assemble the microcontroller, serial board, Arduino Leonardo and interface.

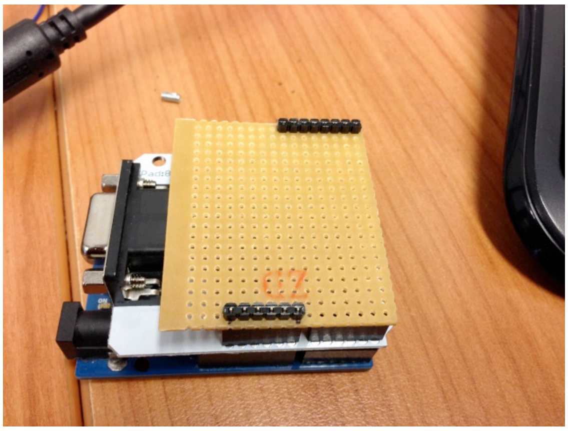

19. Place the assembly in a plastic box and put the cover on for the DJhero

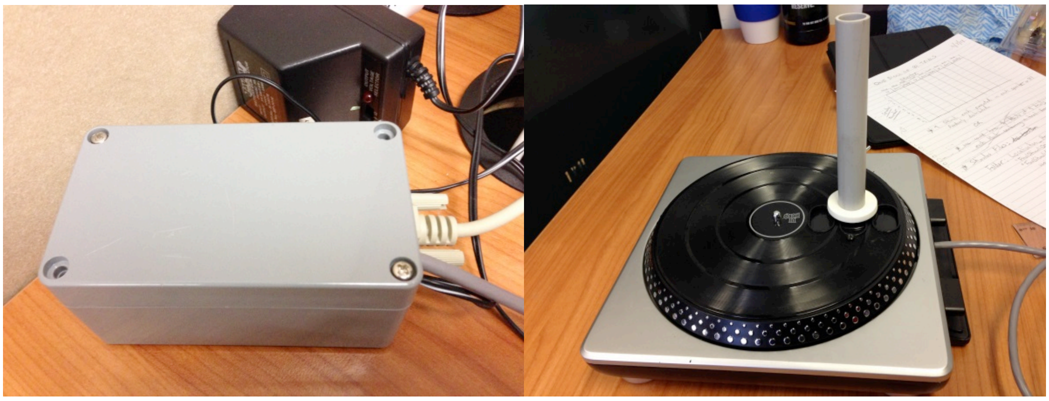

Supplement: SI_2_Instruction for building a music box using a DJ hero_.pdf [file rsos150286supp2.pdf]
